# Biomethanation of Syngas Using Anaerobic Sludge: Shift in the Catabolic Routes with the CO Partial Pressure Increase

**DOI:** 10.3389/fmicb.2016.01188

**Published:** 2016-08-03

**Authors:** Silvia Sancho Navarro, Ruxandra Cimpoia, Guillaume Bruant, Serge R. Guiot

**Affiliations:** ^1^Bioengineering Group, Energy, Mining and Environment, National Research Council CanadaMontreal, QC, Canada; ^2^Department of Microbiology, Infectiology and Immunology, Université de MontréalMontreal, QC, Canada

**Keywords:** syngas, carbon monoxide, anaerobic, methanation, carboxydotrophic methanogenesis, syntrophic acetate oxidation

## Abstract

Syngas generated by thermal gasification of biomass or coal can be steam reformed and purified into methane, which could be used locally for energy needs, or re-injected in the natural gas grid. As an alternative to chemical catalysis, the main components of the syngas (CO, CO_2_, and H_2_) can be used as substrates by a wide range of microorganisms, to be converted into gas biofuels, including methane. This study evaluates the carboxydotrophic (CO-consuming) methanogenic potential present in an anaerobic sludge from an upflow anaerobic sludge bed (UASB) reactor treating waste water, and elucidates the CO conversion routes to methane at 35 ± 3°C. Kinetic activity tests under CO at partial pressures (p_CO_) varying from 0.1 to 1.5 atm (0.09–1.31 mmol/L in the liquid phase) showed a significant carboxydotrophic activity potential for growing conditions on CO alone. A maximum methanogenic activity of 1 mmol CH_4_ per g of volatile suspended solid and per day was achieved at 0.2 atm of CO (0.17 mmol/L), and then the rate decreased with the amount of CO supplied. The intermediary metabolites such as acetate, H_2_, and propionate started to accumulate at higher CO concentrations. Inhibition experiments with 2-bromoethanesulfonic acid (BES), fluoroacetate, and vancomycin showed that in a mixed culture CO was converted mainly to acetate by acetogenic bacteria, which was further transformed to methane by acetoclastic methanogens, while direct methanogenic CO conversion was negligible. Methanogenesis was totally blocked at high p_CO_ in the bottles (≥1 atm). However it was possible to achieve higher methanogenic potential under a 100% CO atmosphere after acclimation of the sludge to CO. This adaptation to high CO concentrations led to a shift in the archaeal population, then dominated by hydrogen-utilizing methanogens, which were able to take over acetoclastic methanogens, while syntrophic acetate oxidizing (SAO) bacteria oxidized acetate into CO_2_ and H_2_. The disaggregation of the granular sludge showed a negative impact on their methanogenic activity, confirming that the acetoclastic methanogens were the most sensitive to CO, and *a contrario*, the advantage of using granular sludge for further development toward large-scale methane production from CO-rich syngas.

## Introduction

Synthesis gas, or “syngas,” produced by thermal gasification of biomass has received increased attention for energy recovery in the past decades due to its higher efficiency compared to other bioenergy processes (McKendry, 2002; Huber et al., 2006). The principal components of syngas, carbon monoxide (CO), carbon dioxide (CO_2_), and hydrogen (H_2_), can serve as substrates for the production of chemicals and fuels, namely methane, through a wide range of microorganisms (Klasson et al., 1991; Sipma et al., 2003; Guiot et al., 2011; Alves et al., 2013; Abubackar et al., 2015). Biomethane can therefore be used to replace natural gas extracted from fossil fuel sources and re-injected into the natural gas grid. However thus far, few microorganisms able to reduce syngas' CO into methane have been discovered (Daniels et al., 1977; Rother and Metcalf, 2004; Oelgeschläger and Rother, 2008; Ferry, 2010). A good source of carboxydotrophic (CO-consuming) microorganisms is anaerobic wastewater-treating sludge, which can be exploited for methane production at large scale (Sipma et al., 2004; Guiot et al., 2011).

The anaerobic conversion of CO can support a variety of microorganisms from different trophic groups within a microbial community. Therefore the pathways involved in methane production from CO become more complex when working with a mixed anaerobic consortium. Carbon monoxide dehydrogenase (CODH) is the enzyme involved in the CO oxidation according to: CO + H_2_O → CO_2_ + 2H^+^ + 2e^−^. This enzyme is present in all carboxydotrophic microorganisms currently known, including methanogens. The oxidation of CO by the CODH provides the energy required to reduce the different substrates in order to produce H_2_, acetate, and methane (Thauer et al., 1977). Recent studies have pointed out that the electron production from CO is thermodynamically favorable in comparison to H_2_, and thus CO can theoretically replace H_2_ as the electron donor in all the microorganisms that contain CODH (Oelgeschläger and Rother, 2008; Hu et al., 2011).

CO can be metabolized by four main trophic groups of microorganisms: methanogenic (methane-producing) archaea, hydrogenogenic (hydrogen-producing) bacteria, acetogenic (acetate-producing) bacteria, and sulfate-reducing bacteria (Mörsdorf et al., 1992; Oelgeschläger and Rother, 2008). Thus, when working with a mixed methanogenic culture it is essential to consider all possible reactions, which are involved in the conversion of CO to methane. Carboxydotrophic methanogenic archaea are able to convert CO directly to methane through the following reaction:

(1) $$4\text{CO}+{\text{2H}}_{2}\text{O}\to {\text{CH}}_{4}+{3\text{CO}}_{2}\;(\Delta \text{G}{}^{\circ}\prime =-212\text{kJ}/\text{reaction})$$

However, methane can also be produced from CO indirectly via other metabolites such asH_2_ and CO_2_ or formate, produced by hydrogenogenic fermentation, followed by hydrogenotrophic methanogenesis, or acetate produced from CO by acetogenic bacteria with subsequent acetoclastic methanogenesis. The main indirect carboxydotrophic methanogenic reactions can be summarized as follows:

(2a) $$\text{CO}+{\text{H}}_{2}\text{O}\to {\text{H}}_{2}+{\text{CO}}_{2}\;(\Delta \text{G}{}^{\circ}\prime =-20\text{kJ}/\text{reaction})$$

(2b) $${\text{CO}}_{2}+4{\text{H}}_{2}\to {\text{CH}}_{4}+{\text{2H}}_{2}\text{O }(\Delta \text{G}{}^{\circ}\prime =-131\text{kJ}/\text{reaction})$$

(2c) $$\text{CO}+3{\text{H}}_{2}\to {\text{CH}}_{4}+{\text{H}}_{2}\text{O }(\Delta \text{G}{}^{\circ}\prime =-151\text{kJ}/\text{reaction})$$

(3a) $$\text{CO}+{\text{H}}_{2}\text{O}\to \text{HCOOH }(\Delta \text{G}{}^{\circ}\prime =-16\text{kJ}/\text{reaction})$$

(3b) $$\begin{array}{l} 4\text{HCOOH}+{\text{H}}_{2}\text{O}\to {\text{CH}}_{4}+{\text{3CO}}_{2}+{\text{3H}}_{2}\text{O}\\ \;(\Delta \text{G}{}^{\circ}\prime =-145\text{kJ/reaction}) \end{array}$$

(4a) $$\begin{array}{l} 4\text{CO}+{\text{2H}}_{2}\text{O}\to {\text{CH}}_{3}\text{COOH}+{\text{2CO}}_{2}\\ \;(\Delta \text{G}{}^{\circ}\prime =-176\text{kJ/reaction}) \end{array}$$

(4b) $${\text{CH}}_{3}\text{COOH}\to {\text{CH}}_{4}+{\text{CO}}_{2}\;(\Delta \text{G}{}^{\circ}\prime =-31\text{kJ/reaction})$$

When combining either the reactions (2a) and (2b), or (2a) and (2c), or (3a) and (3b), or (4a) and (4b), the net production of methane from CO occurs in all cases as follows:

(5) $$4\text{CO}+{\text{2H}}_{2}\text{O}\to {\text{CH}}_{4}+{\text{3CO}}_{2}$$

In addition, homoacetogenic bacteria can participate in the conversion of the H_2_ and CO_2_ to acetate, a thermodynamically favorable reaction, as follows:

(6) $$\begin{array}{l} 4{\text{H}}_{2}+{\text{2CO}}_{2}\to {\text{CH}}_{3}\text{COOH}+{\text{2H}}_{2}{\text{OG}}^{{}^{\circ}}\prime \\ \;(\Delta {\text{G}}^{{}^{\circ}}\prime =-104\text{kJ/reaction}) \end{array}$$

Conversely, syntrophic acetate-oxidizing (SAO) bacteria can convert acetate to H_2_ and CO_2_ when acetoclastic methanogenesis (reaction 4b) is deficient (Karakashev et al., 2006), as follows:

(7) $${\text{CH}}_{3}\text{COOH}+{\text{2H}}_{2}\text{O}\to {\text{4H}}_{2}+{\text{2CO}}_{2}(\Delta \text{G}{}^{\circ}\prime =+95\text{kJ}/\text{reaction})$$

The SAO reaction becomes thermodynamically favorable at low H_2_ partial pressure (< 10^−4^ atm at 35°C) (Lee and Zinder, 1988; Cord-Ruwisch et al., 1998). Moreover, some carboxydotrophic bacteria are able to convert CO into other metabolites such as ethanol, propionate, butyrate, and butanol, as follows:

(8) $$\begin{array}{l} 6\text{CO}+{\text{3H}}_{2}\text{O}\to {\text{CH}}_{3}{\text{CH}}_{2}\text{OH}+{\text{4CO}}_{2}\\ \;(\Delta \text{G}{}^{\circ}\prime =-222\text{kJ}/\text{reaction}) \end{array}$$

(9) $$\begin{array}{l} 7\text{CO}+{\text{3H}}_{2}\text{O}\to {\text{CH}}_{3}{\text{CH}}_{2}\text{COOH}+4{\text{CO}}_{2}\\ \;(\Delta \text{G}{}^{\circ}\prime =-308\text{kJ/reaction}) \end{array}$$

(10) $$\begin{array}{l} 10\text{CO}+4{\text{H}}_{2}\text{O}\to {\text{CH}}_{3}{\left({\text{CH}}_{2}\right)}_{2}\text{COOH}+6{\text{CO}}_{2}\\ \;(\Delta \text{G}{}^{\circ}\prime =-440\text{kJ}/\text{reaction}) \end{array}$$

(11) $$\begin{array}{l} 12\text{CO}+5{\text{H}}_{2}\text{O}\to {\text{CH}}_{3}{\left({\text{CH}}_{2}\right)}_{3}\text{OH}+8{\text{CO}}_{2}\\ \;(\Delta {\text{G}}^{{}^{\circ}}\prime =-480\text{kJ}/\text{reaction}) \end{array}$$

All the above products can then be converted into methane indirectly via acetate and H_2_/CO_2_ (Mazumder et al., 1985; Liou et al., 2005; Henstra et al., 2007).

To enhance the carboxydotrophic methanogenic potential of natural mixed anaerobic cultures and to further facilitate the reactor's scale-up and optimization, it is important to understand the prevalent metabolic pathways of the microorganisms and how the metabolism is affected by environmental conditions. To address this issue this study is focused on the assessment of the carboxydotrophic methanogenic potential present in an anaerobic wastewater-treating sludge from an upflow anaerobic sludge bed (UASB) reactor as well as the identification of CO conversion routes to methane under mesophilic temperatures with the use of specific metabolic inhibitors for bacteria and archaea (methanogens). The experimental design includes an evaluation of the impact of variables such as the substrate concentration and the biomass morphology, on the carboxydotrophic methanogenic potential of the sludge.

## Materials and methods

### Sludge

The tests carried out for this study were performed under mesophilic conditions (35°C), using anaerobic granular sludge from a full-scale upflow anaerobic sludge blanket (UASB) plant treating fruit processing wastewater (Lassonde Inc., Rougemont, QC, Canada). To disaggregate the granules, the sludge was sieved using a mesh grid with 0.25-mm openings and crushed with a mortar under a N_2_ atmosphere. The sieved–crushed sludge was resuspended in 0.05 M phosphate buffer at pH 7.5.

### Experimental design

#### Identification of methanogenic carboxydotrophic potential and toxicity

Carboxydotrophic and methanogenic specific activity tests were performed in triplicate on the suspended anaerobic sludge as well as on whole granules. The tests were carried out with CO as the sole substrate in 60 mL serum bottles. The bottles were filled with 20 mL of the inoculum diluted with 0.05 M phosphate buffer at pH 7.5 to an initial concentration of 2 g volatile suspended solids (VSS)/L. To establish anaerobic conditions the bottles were capped, sealed with butyl rubber stoppers and flushed with N_2_ gas (100%) for 3 min. Afterwards the CO was injected into the bottles under anaerobic conditions. The CO partial pressure (p_CO_) ranged between 0.1 and 1.5 atm (20–100% CO, N_2_ balance), and corresponded to CO concentrations in liquid varying from 0.09 to 1.31 mM, using a value of 1148 atm.L/mol for the Henry constant at 35°C in a buffered media (Zhao et al., 2013). For the tests at a p_CO_ lower than 1 atm, a volume of N_2_ is removed from the bottle and replaced by an equivalent volume of CO using a gas tight syringe to obtain the required p_CO_ in the headspace. For the test at 1 atm of CO or more, all headspace N_2_ is replaced with CO using the manometer of the CO cylinder line to adjust the microcosm headspace to the desired p_CO_. The bottles were immediately placed in dark environmental conditions in a rotary shaker (New Brunswick, Edison, NJ) controlled thermostatically at 35 ± 3°C and operated at 200 rpm to maximize the gas-liquid mass transfer. During the incubation period the bottles' headspace was sampled for CH_4_,H_2_ and CO analysis by gas chromatography at regular time intervals depending on the initial CO concentration until the CO was totally depleted. From the chromatogram, the volume for each gas species measured was computed into mmol per bottle, knowing the temperature of the sample, the volume of injection at standard pressure and the volume of the bottle's headspace. The gas dissolved in the liquid has been neglected as it represents less than 1.5% of the total amount. The bottles' gas content-time curves were drawn using a smoothing curve fit function (KaleidaGraph 4.5, Synergy Software, Reading, PA), which applies the Stineman algorithm to the data (Stineman, 1980). The output of this function has a geometric weight applied to the current point and ±10% of the data range, to arrive at the smoothed curve. The maximum CO consumption or CH_4_ production rates were calculated by a least-squares-based linear regression over three to six values around the inflection point of the CO depletion and CH_4_ accumulation time-courses. The specific activity was obtained by dividing the consumption or production rate by the VSS content in the serum bottle, and expressed in mmol of CO or CH_4_ per gram of VSS per day, as formerly described (Guiot et al., 1995, 2011). At the end of each assay liquid samples from each bottle were analyzed for the presence of volatile fatty acids (VFA) and alcohols.

Four control tests were also performed: an endogenous activity test (without substrate), an inhibited endogenous activity test (with cyanide), a negative control (with CO and cyanide), and lastly an abiotic test (with a basal medium without sludge).

#### Identification of possible routes to methane

**Figure 1:**
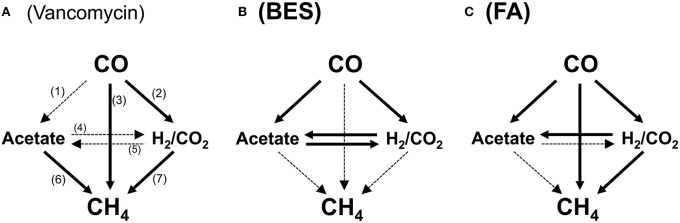
**Possible catabolic routes from CO to methane in presence of vancomycin (A), 2-bromoethane sulfonate (BES) (B), and fluoroacetate (FA) (C)**. The solid arrows indicate the pathways available in presence of the inhibitor; the dotted arrows indicate pathways blocked in presence of the inhibitor, totally or partially, in the case of vancomycin, as gram negative bacteria remain active. Pathways: (1), carboxydotrophic acetogenesis; (2), carboxydotrophic hydrogenogenesis; (3), carboxydotrophic methanogenesis; (4), syntrophic acetate oxidation; (5), homoacetogenesis; (6), acetoclastic methanogenesis; (7), hydrogenotrophic methanogenesis.

To identify the actual routes for CO conversion to methane, assays were carried out with inhibitors. The tests were performed in the same manner as described above, however the bottles were injected at the start of the test, prior to incubation, with the specific metabolic inhibitors: 50 mM 2-bromo-ethane sulfonic acid (BES) (sodium salt, 98% purity, Sigma-Aldrich, Netherlands), used as a methanogenic inhibitor, 5 mM fluoroacetic acid (FA) (sodium salt, 98% purity, Sigma-Aldrich, USA), used as inhibitor of the acetate catabolism (Banat et al., 1981; Chidthaisong and Conrad, 2000), or 0.07 mM vancomycin (hydrochloride hydrate, Sigma-Aldrich, USA) inhibitor of gram-positive bacteria (Nicklin et al., 1999), generally including acetogenic bacteria. The specific concentrations of the inhibitors were chosen from metabolic studies in the literature on the efficiency of the inhibitory effect on the desired activity (Banat et al., 1981; Bagley and Gossett, 1990; Sipma et al., 2004; Sancho Navarro et al., 2014). All of the inhibitory tests were carried out in duplicate. The impact of the three inhibitors mentioned above on the different pathways of CH_4_ production from CO are graphically summarized in Figure 1.

#### Effect of a long-time exposure of the anaerobic sludge to CO

To evaluate the effect of a long-time exposure to high CO concentrations on the carboxydotrophic and methanogenic microbial populations, further activity tests were carried out similarly as described above. For that purpose the disaggregated sludge was incubated during 45 days with continuous CO injections in the headspace, creating an atmosphere of 100% CO. In addition, denaturing gradient gel electrophoresis (DGGE) experiments were performed in parallel to examine changes in the microbial community structure over the time. Samples for DGGE analyses were taken from the bottles after one month and at the end of the 45 days incubation period.

### Genomic analyses

Total genomic DNA was extracted from 2 mL homogenized sludge samples as previously described (Lévesque et al., 1997; Tresse et al., 2002), and then purified and concentrated using a QIAEX gel extraction kit (Hoffman-La Roche AG, USA) according to the manufacturer's instructions. DGGE experiments were performed as previously described (Tresse et al., 2005). Briefly, 16S rDNA sequences were amplified using the primers 341f (5′-CCTACGGGAGGCAGCAG-3′) (Muyzer et al., 1993) and 758r (5′-CTACCAGGGTATCTAATCC-3′) (Lee et al., 1993) for Eubacteria, and the primers 931f (5′-AGGAATTGGCGGGGGAGCA- 3′) (Einen et al., 2008) and 1392r (5′- ACGGGCGGTGTGTAC - 3′) (Kozubal et al., 2008) for Archaea. After electrophoresis, bands of interest were excised from the gel, re-amplified, and submitted to sequencing (Université Laval, Québec, QC, Canada). The sequences were analyzed and then compared to those in the GenBank database using the Basic Local Alignment Search Tool (BLAST) at the National Center for Biotechnology Information (NCBI) to determine the phylogenetic affiliations.

### Analytical methods

The gas components (O_2_, H_2_, CH_4_, N_2_, CO, CO_2_) were determined by gas chromatography. Gas sample of 300 μL (model 1750 gas-tight syringe, Hamilton, Reno, NV) was injected on an Agilent 6890 gas chromatograph (Wilmington, DE) equipped with a TCD and a 5 m × 2.1 mm Carboxen-1000 column (Supelco, Bellafonte, PA) with argon as a carrier gas. The column temperature was held at 60°C for 7 min and increased to 225°C at a rate of 60°C per min. Volatile fatty acids (VFA) (acetate, propionate, and butyrate) and alcohols (methanol, ethanol, acetone, 2-propanol, tert-butanol, n-propanol, sec-butanol, and n-butanol) were measured on an Agilent 6890 gas chromatograph (Wilmington, DE) equipped with a flame ionization detector (FID) as described previously (Guiot et al., 2011). The volatile solids (VS), VSS, and chemical oxygen demand (COD) analyses were performed according to standard methods (Eaton et al., 1995).

## Results

### Carboxydotrophic methanogenic potential

**Figure 2:**
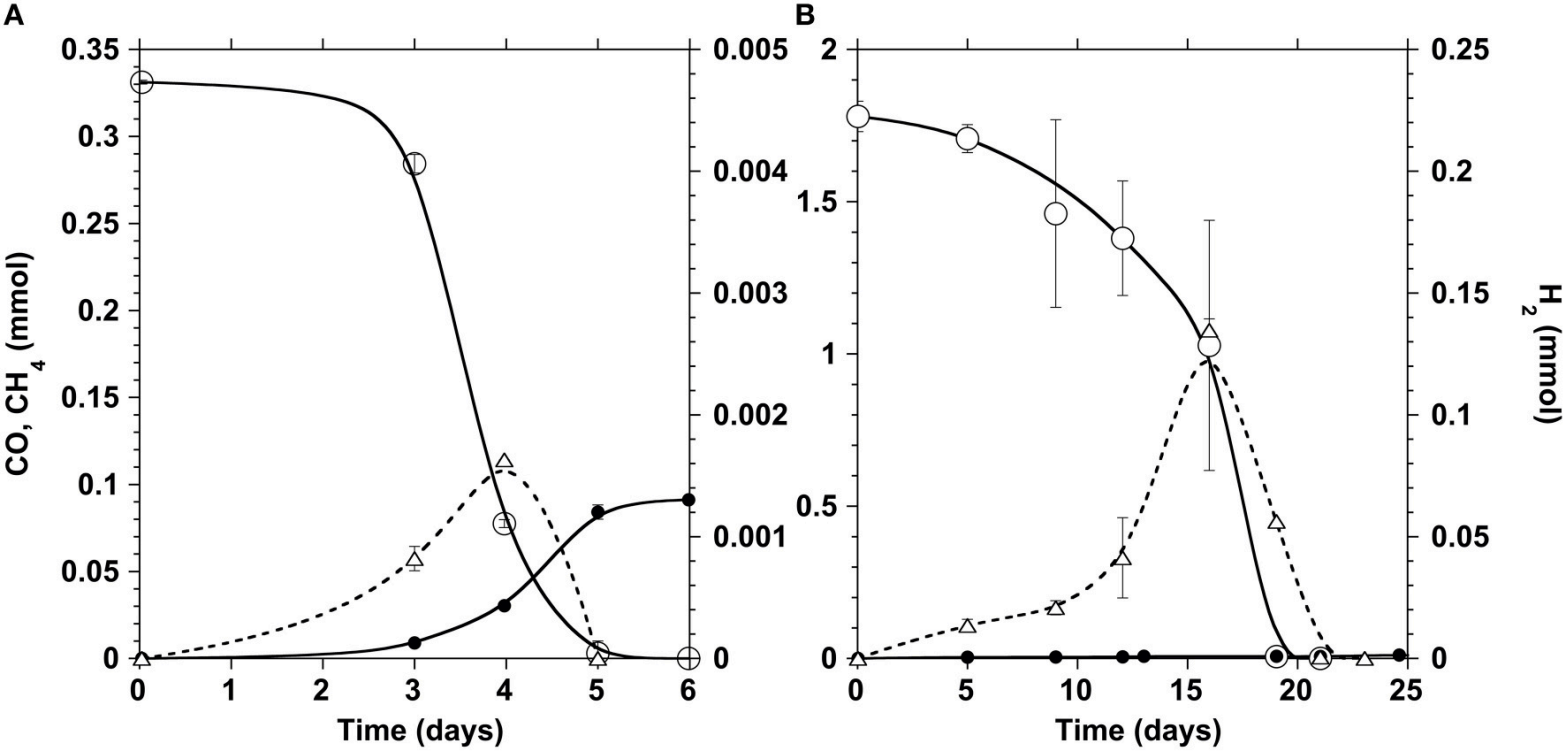
**Time courses of CO (open circles), CH_4_ (filled circles), and H_2_ (triangles) in the incubation bottles at a p_CO_ of 0.2 (A) and 1 atm (B)**. The ordinate values refer to the whole gas content of test bottles.

The disaggregated anaerobic sludge granules were first characterized for their carboxydotrophic and methanogenic potential at different CO partial pressure (p_CO_) in the gas phase. Typical time courses of CO depletion and hydrogen and methane production at a p_CO_ of 0.2 atm are shown in Figure 2A. Although the sludge was non-adapted, the lag time observed was short at low p_CO_, and complete CO depletion occurred within two days_._ Generally accumulation of H_2_ was detected in the bottles, achieving highest H_2_ concentrations when the carboxydotrophic activity was maximal, after which hydrogen declined while the methane production rate appeared to increase.

Since CO is known to act as an inhibitor of methanogenesis, a kinetic activity test was performed to define the optimal CO concentration required to achieve maximum carboxydotrophic and methanogenic activities. The tests were carried out under different initial partial pressures of CO varying from 0.1 to 1.5 atm (0.09–1.31 mmol/L in the liquid phase), at mesophilic conditions (35 ± 3°C) (Table 1). The carboxydotrophic activities observed ranged between 1.8 and 8.6 mmol CO consumed per g VSS per day. The CO activity increased with the amount of CO supplied, and reached its maximum at a p_CO_ of 0.5 atm in the gas phase (0.44 mM in the liquid phase). Beyond p_CO_ of 0.5 atm carboxydotrophic activity declined and there was a significant lag phase prior to CO consumption (Figure 2B). The methanogenic activity from CO evolved differently, being at a maximum (around 1 mmol/g VSS.d) up to a p_CO_ of 0.2 atm (0.17 mM). Then the methane production rate decreased with the increase in CO concentration, until it was totally blocked at a p_CO_ of 1 atm (0.87 mM).

**Table 1:** **Carboxydotrophic and methanogenic activities and product yields of the disaggregated anaerobic sludge granules as a function of the CO concentration in the liquid phase at 35°C**.

CO initial content mmol/L^*^ (p_CO_, atm)	*t*_max_^**^ (d)	**CO specific activity (mmol CO/g VSS.d)**	**CH_4_ specific activity (mmol CH_4_/g VSS.d)**	**Products formed (% of the stoichiometric yield)^‡^**			**H_2_ at maximum (atm.10^−3^)**
				**CH_4_**	**Acetate**	**Propionate**	
0.09 (0.10)	4	1.77 ± 0.20	1.17 ± 0.15	132 ± 2	9 ± 7	21 ± 22	0.5 ± 0.06
0.17 (0.20)	3	5.37 ± 0.26	0.99 ± 0.02	95 ± 5	5 ± 3	7 ± 0.3	1.1 ± 0.02
0.26 (0.30)	5	7.13 ± 1.27	0.54 ± 0.02	62 ± 38	38 ± 18	9 ± 0.2	2.4 ± 2.8
0.44 (0.50)	6	8.62 ± 0.89	0.45 ± 0.08	24 ± 3	47 ± 5	9 ± 1	3.7 ± 1.2
0.87 (1.0)	14	7.65 ± 1.12	0.05 ± 0.03	2 ± 0.5	46 ± 18	21 ± 9	56 ± 13
1.31 (1.5)	37	7.09 ± 0.57	0.00 ± 0.00	0.5 ± 0.1	33 ± 24	15 ± 12	50 ± 5

* *Dissolved CO concentrations from 0.09 to 1.31 mmol/L, are calculated using a value of 1148 atm·L/mol for the Henry constant at 35°C (Zhao et al., 2013)*.

** *Time to reach maximum activity*.

‡ *Stoichiometric yields: ^1^/_4_ mol of CH_4_ per mol CO (reaction 1 or 5); ^1^/_4_ mol of acetate per mol CO (reaction 4a); 1/7 mol of propionate per mol CO (reaction 9)*.

Methane, acetate, propionate, and H_2_ were the main products of the CO conversion, and their yield varied depending on the initial CO concentration (Table 1). High concentrations of CO clearly affect the CH_4_ yield as has been reported in previous studies with anaerobic sludge and pure cultures (Sipma et al., 2003; Oelgeschläger and Rother, 2008, 2009). When methanogenesis starts to decrease, the methane precursors (acetate, propionate and H_2_) begin to accumulate, proportionally to the increase of dissolved CO concentration. Both acetogenesis and hydrogenogenesis were still present at concentrations of 1.31 mmol CO/L (p_CO_ 1.5 atm), although their product yield started to decline at CO concentrations higher than 0.87 mmol CO/L (p_CO_ 1 atm).

The optimal conditions observed for CO conversion at a p_CO_ of 0.5 atm in the headspace and higher with the disaggregated sludge significantly differed from previously reported results obtained on integral sludge granules (Guiot et al., 2011). These authors reported a maximum CO consumption rate of 8.1 mmol CO/g VSS.d at 0.2 atm CO initial partial pressure (0.17 mM), and above this value the activity dropped drastically to 2-3 mmol CO/g VSS d. This discrepancy of the results between the present study, performed on disaggregated granular sludge, and previous results obtained with aggregated granular sludge, led us to question the effect of the sludge structure on carboxydotrophic activity and metabolic pathways. Further analysis was therefore conducted through activity tests using aggregated sludge granules from the current study under the same conditions as discussed above, to describe more precisely the impact of the sludge morphology on CO conversion.

**Figure 3:**
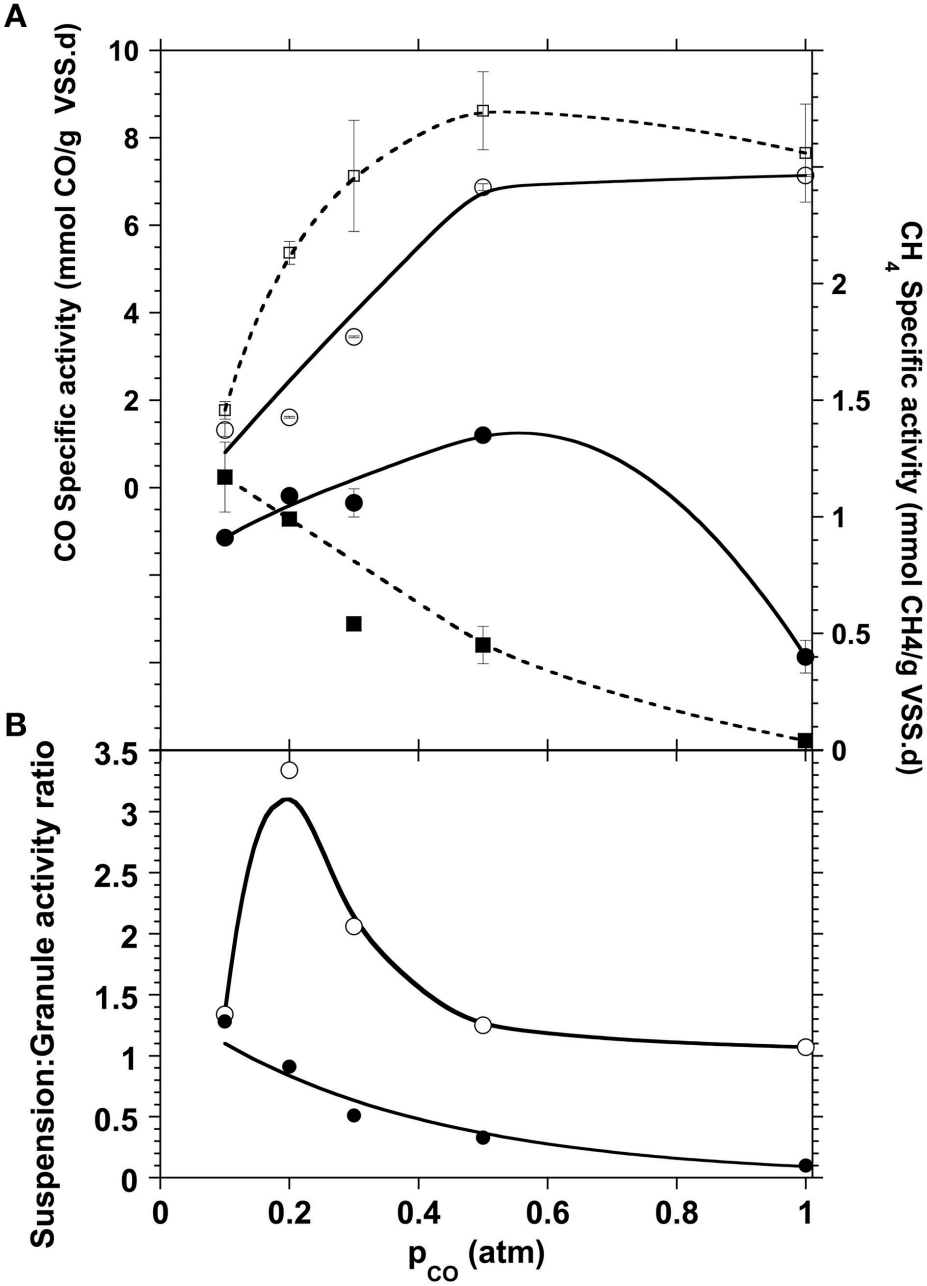
**(A)** Carboxydotrophic (open symbols) and methanogenic (filled symbols) specific activities compared for both disaggregated (squares, dotted lines) and granular (circles, solid lines) sludge morphologies. **(B)** Activity ratio of disaggregated sludge suspension over whole granules for both carboxydotrophic (open circles) and methanogenic (black circles) specific activities.

The experimental results obtained followed the same pattern for the CO conversion (Figure 3). The carboxydotrophic activity increased with the amounts of CO supplied reaching a maximum of 6.9 mmol CO/g VSS.d at a partial pressure of 0.5 atm CO in the gas phase (0.44 mM). In general the CO consumption rate in granular morphology was slightly slower than in the disaggregated granules, which might be explained by the lower CO and metabolic intermediates diffusion within granules and respectively, the lower substrate availability for the carboxydotrophic microorganisms present within the granule inner layers. The methane production pattern also differed depending on the morphology of the sludge. With the whole sludge granules, the methane production reached a maximum rate of 1.15 mmol CH_4_/g VSS.d at a p_CO_ of 0.5 atm (0.44 mM), before to decrease at higher partial pressure, while that of the disaggregated sludge suspension started to decrease at a p_CO_ as low as 0.2 atm (Figure 3). Moreover, even if the methane yield decreased with the increase of the CO concentration independently of the sludge morphology, this decrease was more pronounced with the disaggregated sludge. When the p_CO_ increased from 0.2 to 1 atm the CH_4_ yield of the disaggregated granules decreased from the stoichiometric maximum to 2%, compared to the methane yield of whole granules, which decreased to 12%.

This negative impact that the disruption of the granule had on methanogenic activities is likely due to the protection that the granule offers to the methanogens. This is related to the layered distribution of the different trophic groups in the granular consortium, as reported in many studies (Guiot et al., 1992; Fang et al., 1994; Hwu et al., 1996; Sekiguchi et al., 1999; Fang, 2000; Diaz et al., 2006). The methanogens that are mostly in the core of the granule are then protected against toxic CO concentrations, allowing the maintenance of the methanogenic potential. Such a morphology also allows for a closer juxtaposition between the different trophic groups of microorganisms that participate in the CO conversion to CH_4_, hence enhancing the flux of metabolites between their producers and consumers.

### CO conversion routes

**Figure 4:**
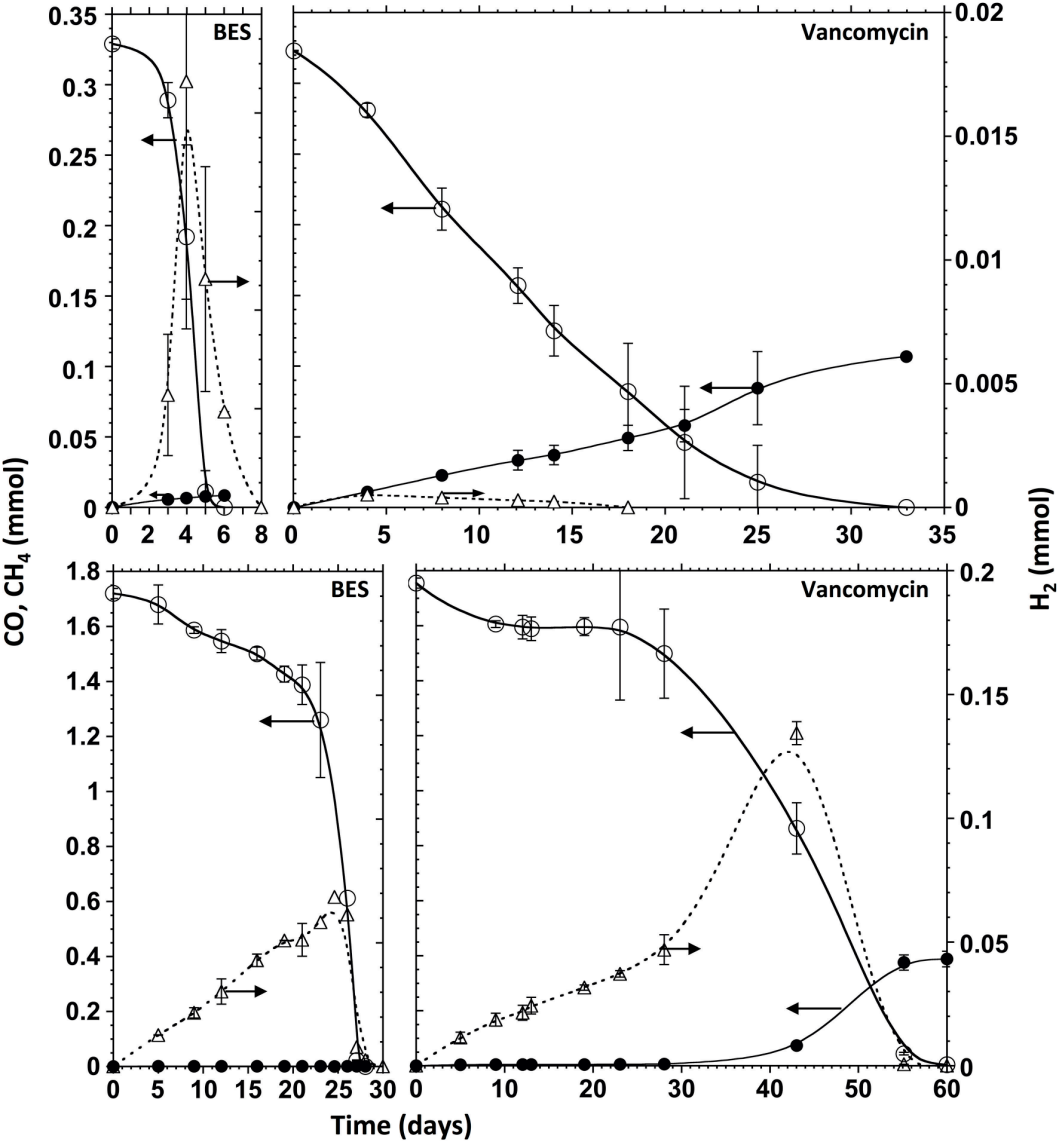
**Time courses of CO (open circles, solid line), CH_4_ (filled circles, solid line) and H_2_ (triangles, dotted line) in presence of BES (left panels), and vancomycin (right panels), at 0.2 atm of CO (upper panels) and 1 atm of CO (lower panels) in the headspace**. The ordinate values refer to the whole gas content of test bottles.

The major metabolites of CO conversion are CH_4_ and acetate, however the presence of hydrogen, and propionate is also noted in the microcosms. These observations suggest that acetate is the main methane precursor. However, to determine the possible routes of methane formation it is essential to discriminate between a direct conversion of CO to methane and an indirect conversion via acetate, and/or H_2_ and CO_2_ (or formate). In order to understand this, specific inhibitors for bacteria and archaea (methanogens) were used. The use of vancomycin as acetogenic inhibitor allows the evaluation of direct and/or hydrogenotrophic pathway through methane formation. BES generally blocks methanogenesis, while FA inhibits acetate consumption by acetoclastic methanogenic archaea and acetate-oxidizing bacteria (Figure 1). A first series of tests were made at 0.2 atm CO partial pressure in the headspace (0.17 mM) corresponding to the optimal methanogenic activity in the sludge studied. The specific activity results are displayed in Table 2, and the time courses for CO, CH_4_, and H_2_ are shown in Figures 2A, 4 (top). In presence of vancomycin CO conversion rates were very low compared to the uninhibited one, but the entire CO was converted to methane, and little volatile acids were observed. In presence of BES the carboxydotrophic activity was reduced approximately by half (from 5.37 to 2.39 mmol CO/g VSS.d), and led to the accumulation of acetate, propionate, and hydrogen as final products from CO. This decrease in carboxydotrophic activity when methanogenesis is blocked does not mean that direct carboxydotrophic methanogenesis is a significant pathway since the rate of CO conversion to methane in presence of vancomycin was a small fraction of that in the control test. Nonetheless, based on the data obtained with vancomycin tests, one possible explanation for the decrease in the CO conversion rate could be the feedback inhibitory effect by the accumulated products from CO (i.e., acetate and/or H_2_) when methanogenesis is blocked. This scenario of substrate conversion inhibition by the accumulated products has been described earlier by various authors working with anaerobic consortia (Yu and Pinder, 1993; Schulz and Conrad, 1996). In absence of methanogenesis acetate was the metabolite with the highest accumulation at all the CO concentrations tested, which lead us to assume that the partial pressure of CO didn't have any effect on the metabolic pathways involved in methane production, and acetate was the main intermediate. In presence of fluoroacetate (FA) the CO conversion rate was further reduced to 29% of that without inhibitors (from 5.37 to 1.57 mmol CO/g VSS.d), and to 66% of the rate attained in the presence of BES, and neither H_2_ nor propionate was detected at the end of the test. Only 29% of the CO was converted to acetate (possibly due to feedback inhibition). It is noteworthy that the methane yield remained relatively high, at 61% of the stoichiometric yield, even though the acetoclastic activity was inhibited. Consequently the methane produced was coming from a different source (i.a. H_2_/CO_2_).

**Table 2:** **Carboxydotrophic and methanogenic activities and product yields of the disaggregated anaerobic sludge granules under a CO pressure in the gas phase of 0.2 atm (0.17 mM in the liquid phase) and inhibitory effect of 2-bromo-ethane sulfonate (BES) (50 mM), vancomycin (0.07 mM), and fluoroacetate (5 mM) at 35°C**.

**Inhibitor**	*t*_max_^*^ (d)	**CO specific activity (mmol CO/g VSS.d)**	**CH_4_ specific activity (mmol CH_4_/g VSS.d)**	**Products formed (% of the stoichiometric yield)^‡^**			**H_2_ at maximum (atm.10^−3^)**
				**CH4**	**Acetate**	**Propionate**	
−	3	5.37 ± 0.26	0.99 ± 0.02	95 ± 5	5 ± 3	7 ± 0.3	1.1 ± 0.02
Vancomycin	^**^	0.36 ± 0.05	0.14 ± 0.01	130 ± 4	21 ± 15	7 ± 0.4	0.3 ± 0.01
BES	4	2.39 ± 0.46	0.01 ± 0.00	9 ± 0.3	51 ± 8	17 ± 0.0	5.6 ± 2.3
Fluoroacetate	3	1.57 ± 0.27	0.33 ± 0.04	61 ± 5	29 ± 0.8	0	0.9 ± 0.2

* *Time to reach maximum activity*.

** *The activity rate did not change for the experiment duration*.

‡ *Stoichiometric yields: 1/4 mol of CH_4_ per mol CO (reaction 1 or 5); 1/4 mol of acetate per mol CO (reaction 4a); 1/7 mol of propionate per mol CO (reaction 9)*.

In presence of vancomycin the CO conversion rates observed with all the CO concentrations tested were very low compared to the uninhibited ones (Table 3). Typical time courses of the CO depletion and the hydrogen and methane production at a 1 atm p_CO_ are shown in Figures 2B, 4 (bottom). The lag time preceding the CO depletion increased in presence of BES, and even more in presence of vancomycin. Generally in absence of active acetogens only a fraction of the carboxydotrophic activity was expressed (between 7 and 30%). The maximum rate (2.11 mmol CO/g VSS.d) was achieved at 1 atm CO partial pressure (0.87 mM), which represented only 30% of the CO conversion rate of the test without inhibitor. These data suggest that direct CO conversion to methane or via H_2_/CO_2_ (or formate) as intermediates were not important pathways in the studied sludge. Moreover as opposed to the uninhibited test, when acetogenic bacteria were inhibited in presence of vancomycin the methanogenic activity in the sludge increased with the amount of CO applied, with a peak of 0.74 ± 0.06 mmol CH_4_/g VSS.d at 1 atm p_CO_, which is not far from the maximum methanogenic activity observed without inhibitor.

**Table 3:** **Carboxydotrophic and methanogenic activities and product yields of the disaggregated anaerobic sludge granules, under different CO initial concentrations in presence of BES (50 mM), and vancomycin (0.07 mM) at 35°C**.

**CO initial liquid conc. (mM) (headspace, atm)**	*t*_max_ (d)^*^	**CO specific activity (mmol CO/g VSS.d)**	**Relative CO conversion rate (%)^§^**	**CH_4_ specific activity (mmol CO/g VSS.d)**	**Relative CH_4_ conversion rate (%)^§^**	**Products formed (% of the stoichiometric yield)^‡^**			**H_2_ at maximum (atm.10^−3^)**
						**CH_4_**	**Acetate**	**Propionate**	
**VANCOMYCIN**									
0.09 (0.1)	^**^	0.26 ± 0.03	15 ± 2	0.15 ± 0.03	13 ± 3	154 ± 6	13 ± 3	30 ± 10	0.4 ± 0.01
0.17 (0.2)	^**^	0.36 ± 0.05	7 ± 1	0.14 ± 0.01	14 ± 1	130 ± 4	21 ± 15	17 ± 0.0	0.3 ± 0.01
0.26 (0.3)	20	0.53 ± 0.08	7 ± 1	0.17 ± 0.0	31	113 ± 3	3 ± 1	12 ± 0	0.4 ± 0.005
0.44 (0.5)	44	0.99 ± 0.06	11 ± 1	0.26 ± 0.04	58 ± 18	89 ± 8	8	7	1.2 ± 0.2
0.87 (1.0)	40	2.11 ± 0.32	30 ± 5	0.74 ± 0.06	1480 ± 60	89 ± 0.2	2.6	3.5	85
1.31 (1.5)	55	1.38 ± 0.05	19 ± 1	0.31 ± 0.06	∞	66 ± 22	7 ± 0.1	5.4 ± 1.9	72 ± 1.2
**BES**									
0.09 (0.1)	5	2.10 ± 0.14	119 ± 8	0.05 ± 0.0	4	28 ± 0.2	67 ± 2	18 ± 3	3.3 ± 0.01
0.17 (0.2)	4	2.39 ± 0.46	45 ± 9	0.01 ± 0.0	1	9 ± 0.3	51 ± 8	7 ± 0.4	5.6 ± 2.3
0.26 (0.3)	5	2.14 ± 0.11	30 ± 2	0.03 ± 0.01	6 ± 2	12 ± 3	82 ± 4	17 ± 1	28 ± 14
0.44 (0.5)	8	4.64 ± 0.30	54 ± 3	0.00 ± 0.0	0	2 ± 0	54 ± 3	10 ± 1	22 ± 5
0.87 (1.0)	21	6.30 ± 0.28	82 ± 12	0.001 ± 0.00	2	0.4 ± 0.0	57 ± 14	16 ± 8	37 ± 5
1.31 (1.5)	31	2.92 ± 0.61	41 ± 9	0.000 ± 0.00	0	0.1 ± 0.1	45 ± 7	11 ± 1	37 ± 12

* *Time to reach maximum activity*.

** *The activity rate did not change over the experiment interval*.

§ *The relative conversion rates are calculated as the percentage of the average rate in the control assays (without inhibitor, Table 1)*.

‡ *Stoichiometric yields: ^1^/_4_ mol CH_4_ per mol CO (reaction 1 or 5); ^1^/_4_ mol acetate per mol CO (reaction 4a); 1/7 mol propionate per mol CO (reaction 9)*.

In presence of vancomycin at low p_CO_, the methane yield was significantly higher than the stoichiometric (i.e., maximal) yield (Table 3, p_CO_ 0.1 – 0.3 atm). This was probably due to the degradation of organic matter brought with inoculation (cell lysis products, microbial secretions, extracellular polymeric substances) (Zhang and Bishop, 2003). The products of that organic matter's fermentation by the vancomycin resistant gram-negative bacteria might afterwards be transformed into CH_4_ thus increasing the observed yield. This contribution is likely weightier under substrate-limiting conditions. This was confirmed with endogenous tests (no substrate) and vancomycin. However, at a 1 atm p_CO_ or above, even though the methane production rate was higher than at lower p_CO_, the methane yield from CO decreased, and H_2_ started to accumulate. This observation is consistent with previous work with *M. barkeri* growing on CO as the sole carbon and energy source (O'Brien et al., 1984). In the study H_2_ formation occurred at p_CO_ higher than 0.2 atm in the gas phase, but methane was the main metabolite at CO concentrations below this value. In another study with *M. acetivorans* (Oelgeschläger and Rother, 2009) the authors discuss that the methane formation rate is not inhibited at high CO concentrations, but the increase in CO partial pressure also leads to the rate increase of other products formed from CO, which could cause a decrease in the final amount of CO converted to methane.

In presence of BES, at all CO concentrations above 0.1 mM, the carboxydotrophic activity was reduced to two- to one-third of that obtained without inhibitor. The inhibition of methanogenesis by BES led to the accumulation of acetate, propionate and hydrogen as final products from CO. Acetate was the metabolite with the highest accumulation at all the CO concentrations tested, which confirms that acetate is the main intermediate of the CO transformation into methane. Therefore both methanogenic pathways, via H_2_/CO_2_ (and/or formate) and via acetate, seem to be co-existing if hydrogenogenic bacteria and hydrogenotrophic methanogens are present in the consortium. However, as previously reported (Sancho Navarro et al., 2014), acetoclastic methanogenesis seems to be the dominant pathway when the conditions are favorable for methanogenesis to happen, instead of the direct carboxydotrophic methanogenesis route.

### Effect of long term exposure to CO

Since the methane production rate observed in the vancomycin assays tend to increase at higher CO concentrations, together with the lag time required for the carboxydotrophic activity to be achieved, this suggests that adaptation of the microbial populations present in the sludge can occur over time due to exposure to the high CO concentrations. To evaluate this hypothesis, activity tests under a fixed 100% CO atmosphere in the gas phase were performed over 45 days. The carboxydotrophic activity and methane potential achieved over time are presented in Table 4. There is a clear correlation between the exposure time to CO and the methanogenic potential in the consortium. Both the carboxydotrophic and methanogenic activity increased drastically between day 30 and 45 of incubation, achieving a maximum methane production rate of 5.5 ± 1.2 mmol CH_4_/g VSS.d at day 45. After such an acclimation of the sludge it was possible to reach a yield of 90% at a p_CO_ of 1 atm, whereas without acclimation, this yield was only 8% for the same CO pressure conditions.

**Table 4:** **Change in the carboxydotrophic and methanogenic rate potential of the disaggregated anaerobic sludge granules, under a CO 100% atmosphere in the gas phase, as a function of the adaptation time**.

**Time days**	**CO specific activity mmol CO/g VSS.d**	**CH_4_ specific activity mmol CH_4_/g VSS.d**	**CH_4_ yield stoichiometric %^‡^**	**Acetate yield stoichiometric %^‡^**	**Accumulated acetate concentration mM**	**Max. p_H2_ atm.10^−3^**
0	8.5 ± 2.7	0.04 ± 0	7.8 ± 0.63	10.3 ± 4.1	0.18	56 ± 13
30	11.7 ± 1.6	0.7 ± 1.3	23.0 ± 44.5	–	–	152 ± 25
45	24.2 ± 5.9	5.5 ± 1.2	90.4 ± 29.6	3.2 ± 0.4	17	43 ± 15

‡ *Stoichiometric yields: 1/4 mol of CH_4_ per mol CO (reaction 1 or 5); 1/4 mol of acetate per mol CO (reaction 4a)*.

To examine the possible variation in the microbial population over the time due to such an adaptation to a 100% CO atmosphere, DGGE experiments were performed in parallel to the activity tests. Sludge was sampled after an incubation of 30 and 45 days. The bacterial and archaeal 16S rDNA sequences of interest were compared either to the GenBank database by using BLAST or to the Ribosomal Database Project (RDP) 16S rDNA database by using the RDP classifier platform. Phylogenetic affiliations determined by BLAST and RDP classifier for the selected bacterial and archaeal 16S rDNA sequences are presented in Table 5. The results confirmed a shift in both the bacterial and the archaeal populations, corresponding to the increased methanogenic potential observed at 45 days of incubation. *Clostridium propionicum*, a propionate producing bacterium (Johns, 1952), and *Acetobacterium wieringae*, an acetate producing bacterium (Braun and Gottschalk, 1982), which were not detected at the beginning of the test, appeared to be important in the bacterial population after a month of incubation at high CO concentrations, suggesting that high p_CO_ conditions positively stimulated their growth. This increase in the abundance of these two species in the microbial community corresponded to the previously observed acetate and propionate accumulation at high CO concentrations in the activity tests. In addition to those two bacteria, *Petrimonas sulfuriphila*, a fermentative acetate and H_2_/CO_2_ producer (Grabowski et al., 2005), and *Geobacter uraniireducens* sp., a SAO bacterium (Shelobolina et al., 2008), were detected after 30 or 45 days of CO exposure. Variations in the archaeal population were also observed, with notably a shift toward a dominance of hydrogen-utilizing methanogens over time. Microorganisms belonging to the orders *Methanomicrobiales* and *Methanobacteriales* were found to be present in the consortium after 30 or 45 days of CO exposure, suggesting a better adaptation of those populations to high CO concentrations.

**Table 5:** **Evolution of the eubacterial and archaeal populations in the anaerobic sludge over the adaptation time, under an atmosphere of 100% CO as determined by DGGE experiments**.

**Identified Microorganism (GenBank Accession Number)**	**% Similarity (Sequence Length)**	**Presence (+) or absence (−) of the microorganism in the population**			**RDP Classifier Classification**
		**Day 0**	**Day 30**	**Day 45**	
**EUBACTERIA**					
*Clostridium propionicum*, strain: JCM 1430 (AB649276.1)	100% (400/400)	−	+	+	*Clostridium* XlVb (100%)
*Acetobacterium wieringae*, strain: DP9 (HQ384240.1)	99% (394/396)	−	+	+	*Acetobacterium* (100%)
Uncultured *Bacteroidetes* bacterium, clone: L D1 16S (HQ003602.1)	99% (357/391)	−	−	+	*Bacteroidetes* (99%)
*Petrimonas sulfuriphila*, strain: BN3 (NR042987.1)	94% (391/415)	−	−	+	*Bacteroidales* (100%), *Petrimonas* (96%)
*Geobacter uraniireducens* Rf4 (CP000698.1)	97% (399/411)	−	+	+	*Desulfuromonadales* (100%), *Geobacteraceae* (97%), *Geobacter* (94%)
*Brevundimonas bullata*, strain: NBRC 13290	98% (380/388)	−	+	−	*Alphaproteobacteria* (100%), *Caulobacteraceae* (99%), *Brevundimonas* (97%)
**ARCHAEA**					
*Methanosaeta concilii*, strain: NBRC 103675 (AB679168.1)	99% (429/434)	+	+	+	*Methanosaeta concilii* (98%)
Uncultured *Methanolinea* sp., clone: SMS-T-Pro-2 (AB479406.1)	99% (431/433)	−	+	+	*Methanolinea tarda* (89%)
Uncultured *Methanobacteriaceae* archaeon, clone: AR-H2-B (AB236069.1)	100% (429/429)	−	−	+	*Methanobacterium congolense* (98%)

## Discussion

Based on the data obtained with the specific inhibitors we assumed that methane production from CO was mainly via acetate as an intermediate metabolite, as previously observed (O'Brien et al., 1984; Sipma et al., 2003; Sancho Navarro et al., 2014). This was further confirmed by the dominance of *Methanosaeta* species in the microbial population, even though the hydrogenotrophic methanogenesis was also present and significant in the sludge examined.

When methanogens were inhibited in presence of BES, acetate was the major metabolite accumulated in all the CO concentrations tested, although H_2_ and propionate were also present but to a lesser extent. In the absence of an inhibitor these metabolites were completely converted to methane under optimal methanogenic conditions (0.2 atm p_CO_), but started to accumulate at higher CO concentrations, due to the inhibitory effect that CO exerts on methanogenesis, as reported in previous work (Oelgeschläger and Rother, 2008, 2009). Other studies (Rother and Metcalf, 2004; Lessner et al., 2006) reported that higher exposure to CO leads to the apparent down-regulation of the operon mtr, which encodes for the enzyme catalyzing the methyl transfer from the N-methyl-tetrahydrosarcinapterin to the coenzyme M, necessary in the last catabolic step before methane production during growth on H_2_/CO_2_ or acetate (Welander and Metcalf, 2005). Hence, at higher CO concentrations when the conditions are unfavorable for methanogens, hydrogen-utilizing bacteria and acetogenic bacteria may take over in the population. These results are consistent with literature, which reports many acetogens and hydrogenogens able to grow at high CO concentrations (Sipma et al., 2006; Oelgeschläger and Rother, 2008; Techtmann et al., 2009).

Methane production from CO via acetate as the main intermediate was further supported in presence of vancomycin when acetogenic bacteria were inhibited. The pronounced decrease in carboxydotrophic activity observed under these conditions clearly indicates that direct methane production from CO or indirectly via H_2_/CO_2_ was secondary in the sludge studied, as has been reported in previous studies (Klasson et al., 1990; Oelgeschläger and Rother, 2008; Guiot et al., 2011; Sancho Navarro et al., 2014). This can be explained by the higher energy balance of CO-utilizing acetogenic bacteria as compared to the carboxydotrophic hydrogenogens (i.e., ΔG°′ = −44 vs. −20 kJ/mol CO), as well as the slightly higher doubling time achieved by hydrogenogens (Henstra et al., 2007). Hence this makes acetogenic bacteria a better competitor for CO than hydrogenogens, the former thus becoming dominant in the population under CO conditions, as was shown in the molecular analyses performed over a long term exposure to CO. On the other hand the minimal direct CO conversion to methane observed in the consortium might be due to the poor kinetic properties of methanogens compared to CO-hydrogenogenic and acetogenic bacteria. This is coherent with previous work where the authors reported a higher CO affinity by the carbon monoxide dehydrogenase (CODH) enzyme in carboxydotrophic hydrogenogens and acetogens than in methanogens (Oelgeschläger and Rother, 2008).

Interestingly, in the vancomycin assays where acetogenic activity was inhibited, an increase of the methanogenic activity with the amount of CO supplied was observed, in contrast to the uninhibited tests. It is hypothesized that the adaptation of methanogens through their longer exposure to CO (longer lag time) allowed for the observed increase. This hypothesis was confirmed with the tests performed under 100% CO, where the sludge achieved the highest methanogenic activity after 45 days of exposure to CO, and reached a 90% CO conversion to methane. Furthermore, the results obtained with the tests performed with long exposure to CO demonstrated that the sludge could achieve higher methanogenic potential under a 100% CO atmosphere through adaptation to CO conditions, possibly through the regulation of the CODH/hydrogenase activity at the molecular level. Once sludge was adapted, 90% of the CO was converted to CH_4_ at the end of the experiment, whereas it was only 8% before acclimation. Previous studies with *M. acetivorans* (Rother and Metcalf, 2004) and *M. barkeri* (O'Brien et al., 1984) demonstrated the microorganisms' ability to grow at 100% CO in the headspace after an adaptation period by stepwise increase of the CO concentration, although the methane production achieved at high CO levels in the gas phase was very low in those studies. Nonetheless, a recent study reported that an isolated *M. acetivorans* strain from prolonged incubations with CO was capable of producing methane directly from CO at high rate (Kliefoth et al., 2012).

Moreover, recent work with *Carboxydothermus hydrogenoformans* describes the regulation of both hydrogenase-linked CODH and CODH/ACS operons for efficient consumption of CO across a wide range of concentrations (Techtmann et al., 2011). Those authors presented that under high p_CO_ the bacteria were able to catabolize more CO into energy by overexpression of the hydrogenase, while at low CO concentrations the CO is mainly used toward carbon fixation. It seems that methanogens needed a longer adaptation time to achieve methane production at high CO concentrations.

The molecular analyses performed in this study showed an adaptation of the microbial population of the sludge to high p_CO_, with an evolution toward dominance of acetate producers and acetate oxidizers. The archaeal population previously dominated by acetoclastic methanogens, namely *Methanosaetaceae* species, evolved into a mixed culture of acetate and hydrogen-utilizing methanogens with dominance of hydrogenotrophic methanogens (*Methanobacteriales* and *Methanomicrobiales*). Several studies already reported that the decrease of *Methanosaetaceae* in the population, related to stressing conditions, such as high acids or ammonia levels, typically leads to the dominance of acetate-oxidizing bacteria in syntrophic cooperation with hydrogen-utilizing methanogens (Schnürer et al., 1999; Karakashev et al., 2005, 2006; Hao et al., 2011; Lü et al., 2016). In addition, other studies (Koster and Lettinga, 1984; Borja et al., 1996; Nozhevnikova et al., 2007) highlight the noticeable sensitivity of acetoclastic methanogens to different by-products in the media, including CO (Bhatnagar et al., 1987; Sipma et al., 2006).

**Figure 5:**
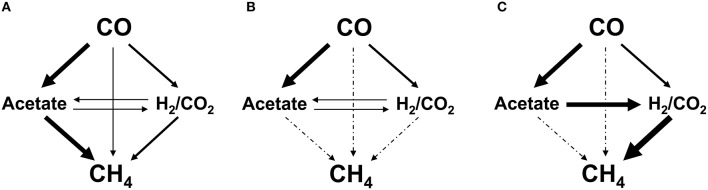
**Suggested pathways for conversion of CO into CH_4_ by a mixed anaerobic sludge, under low p_CO_ (< 0.5 atm) (A), under high p_CO_ (≥1 atm) (B), and after acclimation of the sludge to high CO concentrations (100% CO in the gas phase) (C)**. The thickness of the arrow is representative of the relative importance of the pathway (thick, 60–80%; intermediate, 20–40%; thin, 5–20%). The dotted lines indicate methane production pathways blocked by high CO concentration (p_CO_ ≥ 1 atm).

Therefore, we can conclude that at low CO concentrations CO is converted mainly to acetate by acetogenic bacteria which is subsequently transformed into methane by acetoclastic methanogens (*Methanosaetaceae*), while at high p_CO_ the methanogenic activity seems to be generally inhibited by the amount of CO applied, as previously discussed (Welander and Metcalf, 2005; Oelgeschläger and Rother, 2008, 2009). However, hydrogen-utilizing methanogens, also present in the mixed anaerobic culture, were more tolerant to CO, allowing for the CO conversion to methane behind hydrogenogenesis, at mid CO concentrations (0.5 atm ≤ p_CO_ < 1 atm). Nonetheless, it was possible to achieve methane production at high p_CO_ (≥ 1 atm) after a sufficient acclimation to CO over time, contrary to what is reported in some studies (Sipma et al., 2003; Oelgeschläger and Rother, 2008, 2009). This adaptation to high CO concentrations led to a shift in the archaeal population, resulting in the dominance of hydrogenotrophic methanogens (*Methanobacteriales* and *Methanomicrobiales*), which increased the sludge hydrogen-consuming potential and allowed for low H_2_ concentrations in the medium. This drove the emergence of a syntrophic acetate oxidizing (SAO) pathway, which was able to take over acetoclastic methanogenesis, and to become the main pathway for methane production through the hydrogenotrophic methanogenesis. The proposed CO conversion routes at low and high p_CO_, prior and after adaptation to high CO concentrations, are presented in Figures 5A–C, respectively.

The disaggregation of the granular sludge showed a negative impact on their methanogenic activity, confirming that the acetoclastic methanogens were the most sensitive to CO, and *a contrario*, the advantage of using granular sludge for further development toward large-scale methane production from CO-rich syngas or syngas biomethanation.

## Author contributions

SS carried over the experiments, processed and interpreted the data, discussed the results and wrote the manuscript first draft. RC participated to the experimental design, to the processing, interpretation and discussion of the data, and reviewed the manuscript. GB lead the molecular biology studies, carried over the interpretation and discussion of the metagenomics results, and reviewed the manuscript. SG lead the project definition, participated to the experimental design and to the interpretation and discussion of the data, corrected and edited the final version of the manuscript.

### Conflict of interest statement

The authors declare that the research was conducted in the absence of any commercial or financial relationships that could be construed as a potential conflict of interest.
